# Widespread neuronal activity related to bimanual coordination in non-human primates: evidence from Fos-like activation during bimanual versus unimanual motor task

**DOI:** 10.1177/26331055251352807

**Published:** 2025-06-30

**Authors:** Yu Liu, Eric M. Rouiller

**Affiliations:** 1 Department of Neuroscience, Section of Medicine, Faculty of Sciences and Medicine, University of Fribourg, Fribourg, Switzerland; 2 Present address: Dept. of Biomedical sciences, University of Minesota Medical School, 1035 University Drive, Duluth, MN, USA

**Keywords:** macaque monkeys, bimanual coordination, motor cortical areas, basal ganglia, striatum, functional neuronal mapping, c-fos, hand control

## Abstract

Electrophysiological data support the notion that spatial and temporal coordination between the forelimbs in primates takes place in a wide network of cortical and subcortical brain structures. However, single neuron electrophysiology is biased towards large, long distance projecting neurons. The aim of the present study was to assess whether the same neural network is involved when small and medium size neurons are considered. To address this issue, neuronal activity with cellular resolution was investigated and quantified using the c-fos mapping technique, targeting small and medium size diameter neurons, in adult non-human primates. Two male macaque monkeys^i^ were trained to perform a reach and grasp drawer task, executed either bimanually (BIM) or unimanually (UNI). Extensive single unit electrophysiological recordings were conducted in these two monkeys over a two-year period, preceding a final terminal c-fos session during which one monkey (Mk-1) performed exclusively the BIM task, while the second monkey (Mk-2) performed the UNI task only (250 trials each). One additional monkey (control Mk-3) did not perform any task. Fos-like immunoreactivity (FLI) was significantly higher in both Mk-1 and Mk-2 in motor brain areas than in the control monkey, demonstrating that motor activity triggered c-fos. Although the overall muscle activity was roughly comparable in both tasks, Mk-1 (BIM) exhibited a clearly stronger FLI than Mk-2 all along the rostrocaudal axis of the primary, supplementary and cingulate motor cortices, as well as the striatum. In contrast, Mk-1 and Mk-2 displayed a comparable FLI in non-motor regions, such as the visual and auditory thalamus. The present study, a very rare c-fos mapping investigation conducted in macaques performing a complex behavioral task, suggests that small and medium size (local) neurons may also contribute to the specific neural activity responsible for precise interlimb coordination, within a network associating motor cortical areas and the basal ganglia.

## Background and Aim

Voluntary movements in primates are controlled mainly by parallel corticospinal commands originating from multiple fronto-parietal cortical areas.^1-5^ Additional corticofugal projections directed to various subcortical targets also play a role in motor control, as well as in the functional recovery following CNS lesion or disease.^6^ A particularly sophisticated aspect of motor control in primates is the specific ability to use both hands in a highly coordinated manner, such as knitting, playing an instrument, lacing shoes, buttoning a shirt, to hammer a nail, etc. Bimanual coordination, both in time and spatial domains, was initially proposed to be under the specific control of the supplementary motor area,^7-11^ before the concept of a shared contribution of multiple bilateral fronto-parietal cortical and subcortical structures within a distributed network was privileged.^12-51^

Several electrophysiological studies in non-human primates investigated which motor cortical areas contain single neurons exhibiting coding properties reflecting possible contribution to the control of coordinated bimanual movements: the primary motor cortex (M1),^18,21,24,26,32,36,52^ the supplementary motor area (SMA),^18,21,24,26,36,52^ the premotor cortex (PM),^19,36^ the cingulate motor areas (CMA),^19,53,54^ the posterior parietal cortex (PPC/AIP),^19,47,50^ as well as in subcortical regions.^33,41,42^ Based on a reach and grasp drawer task executed by monkeys either unimanually or bimanually, a proportion of recorded neurons ranging from about 50% to 75% were classified as “bimanual” neurons in M1, SMA, PMd, CMA, and PPC.^18,19^ “Bimanual” neurons were defined as single units for which “their action potential activity observed in bimanual trials could not be predicted from the activity associated with unimanual trials when comparing the same events related to the same arm”, thus suggesting the presence of a bimanual synergy underlying interlimb cooperation. The proportion of “bimanual” neurons was somewhat lower in the striatum (about 40%)^33^ than in the motor cortical areas M1, SMA, PMd, CMA, and PPC.

Single unit electrophysiological recordings conducted in the studies mentioned above are however biased towards large diameter cortical cells, mostly pyramidal cells in cortical layers III and V, corresponding to long distance projecting neurons. The contribution of medium and small diameter cells, involved mostly in local circuits (e.g. interneurons), to the control of bimanual coordinated movements remains largely unknown. The goal of the present investigation was to address this issue based on a cellular functional mapping technique biased towards medium and small cells, taking advantage of the activity related production of the protein Fos by the c-fos immediate early gene. C-fos can be employed as a functional marker with cellular resolution,^55-57^ in particular in response to a variety of external stimulations,^58-64^ as well as in relation to motor activity.^65-71^ A bias of the c-fos mapping technique towards small and medium size cells has been reported, for instance in the auditory system.^60-64^

Based on an original c-fos investigation conducted on behaving adult macaque monkeys, this study aimed to test the hypothesis that activation of small and medium size neurons occurs in brain areas as widespread as those of large, long-distance-projecting neurons in relation to the performance of a bimanual motor coordination task. The same monkeys were the object of a previous electrophysiological investigation in which single unit activity was monitored while performing the same motor sequence executed either with one hand or with both hands.^18,19^

## Methodology

In previously published studies from our laboratory, the functional c-fos mapping method was extensively used in order to investigate the neuronal activation elicited by various modes of stimulation in the auditory system^60,72-76^ or as a result of movement activity in the motor pathways.^18,66^ A comprehensive description of the immunohistological methods to reveal c-fos immunoreactivity on histological sections can be found in the above cited studies. In the three monkeys reported in the present study, Fos-like immunoreactivity (FLI) was revealed using an antibody against Fos provided by Santa Cruz Biotechnology (www.scbt.com; rabbit polyclonal IgG, c-Fos (4), SC-052, lot #D245).

The present study involved three adult male macaque monkeys (macaca fascicularis):

- Monkeys 1 and 2, in which Fos-like activity was assessed in relation to the bimanual task versus the unimanual task, respectively (see below). They were about 5 years old at the time of the terminal c-fos experiment.
- Monkey 3, used as control, in which baseline Fos-like activity was assessed in absence of specific behavioral task performance. The age of Mk-3 at euthanasia was comparable to that of Mk-1 and Mk-2.

**Figure 1.**
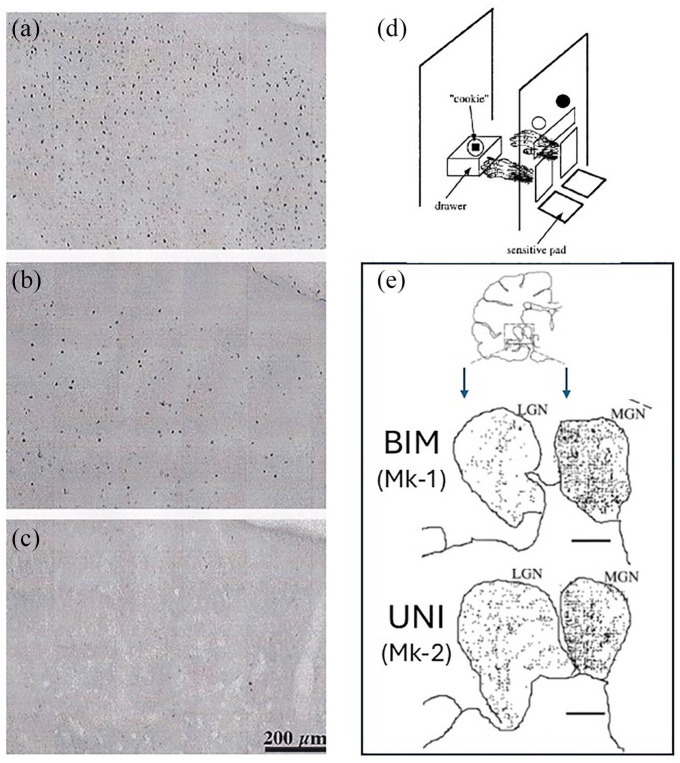
Typical appearance of FLI on photomicrographs of histological sections in the cingulate motor cortex (CMA), in the form of dark dots representing Fos positive cells in Mk1 (a; bimanual task), in Mk-2 (b; unimanual task) and in Mk-3 (c; control - no task). The precise location of the cortical zone depicted in each panel is indicated by the squares pointed by the arrows in Figure 2a, in the right CMA of the 3 monkeys. In (d), the reach and grasp drawer task is represented schematically for a bimanual trial, with a vertical board facing the monkey, comprising two windows each with a sliding door, above two resting pads detecting the presence or absence of the hand on it. The monkey self-initiates a trial by placing each hand on the corresponding resting pad (left and right). Then, two LEDs higher up on the vertical board are used to indicate the monkey whether the coming trial is bimanual (2 LEDs turned on; both windows open), or unimanual left (left LED only turned on; left window open only), or unimanual right (right LED only turned on; right window open only), instructed in a pseudo-random order, as this was the case for the electrophysiological investigations conducted on these 2 monkeys.^18,19^ In contrast, in the present c-fos terminal session, Mk-1 performed only the bimanual task (250 trials), while Mk-2 performed the unimanual left task only (250 trials). A transparent rectangle window provides vision for the monkey to see a drawer placed on a second vertical board in the back. The drawer includes a hole in which a solid reward (“cookie”) was placed automatically by the system before each trial. In the bimanual task (Mk-1), after simultaneous opening of the two windows, the monkey reached the knob of the drawer, pulled it and maintained it open with the same left hand as the drawer was spring loaded; the right hand was used to grasp the reward inside the drawer and transport it back to the mouth. In the left unimanual task (Mk-2), after pulling with the left hand, the drawer was automatically fixed by the system in the open position (neutralizing the effect of the spring load) so that the same left hand was used to grasp the reward, while the right hand had to stay still at all time during the trial in contact with the right resting pad. See Kermadi et al.^17-19^ for more details on the task, set-up, monkey training, etc. In panel E, reconstruction of individual frontal histological sections at the level of the thalamus (top drawing) in the zone comprising the lateral geniculate nucleus (LGN) and the medial geniculate nucleus (MGN) in Mk-1 and Mk-2. In LGN and MGN, positive Fos cells are indicated by dots. FLI was denser in MGN than in LGN. More importantly, the density of FLI in LGN and MGN was comparable in the 2 monkeys, irrespective of the task executed by Mk-1 (bimanual task) or Mk-2 (unimanual task). Scale bars = 1 mm.

Monkeys 1 (Mk-1) and 2 (Mk-2) were trained during 3-6 months to perform a reach and grasp drawer task^17-19^ (Figure 1d; video sequence 1), derived from an earlier version of a comparable bimanual task.^77^ Using either the left hand alone, or the right hand alone, or both hands in a coordinated manner in space and time, Monkeys 1 and 2 were trained to retrieve a reward (food pellet) from a drawer (video sequence 1 in supplemental material). A more advanced version, but comparable, of the unimanual sequence of the reach and grasp drawer task can be visualized on a video sequence in Schmidlin et al.^78^ Although handedness of Mk-1 and Mk-2 was not determined quantitatively with specific batteries of motor tests at that time, they both spontaneously chose to perform the bimanual trials using the right hand to grasp the pellets in the drawer’s slot, suggesting thus a preference for the right hand. Single unit recordings were derived from multiple cortical areas^18,19^ while Monkeys 1 and 2 performed this behavioral paradigm during daily sessions (about 60 minutes), conducted during nearly two years (see video sequence 2 in supplemental material). The temporal sequence of left-unimanual, right unimanual or bimanual trials was determined pseudo-randomly, with the aim to collect a roughly comparable number of the 3 trials types (30-50 trials in total for each recorded single unit). After completion of the electrophysiological investigations, in a very last terminal session without electrophysiological recording, MK-1 performed exclusively the bimanual task during 45 minutes (about 250 trials overall) while Mk-2 performed exclusively the unimanual task with the left hand only (also about 250 trials in about 45 minutes). Then, both monkeys rested for 60 minutes in a quiet and dim room in their primate chair, before they were euthanized with an overdose of pentobarbital (60 mg/kg, i.p.), as previously reported.^17-19^ The additional control monkey (Mk-3), involved in a separate neuroanatomical tracing experiment, was euthanized in a similar way, except that Mk-3 did not perform any particular motor task before administration of the lethal dose of pentobarbital. Mk-3 thus represented a control animal in order to establish a baseline of FLI in absence of intense voluntary movements preceding the euthanasia.

**Figure 2.**
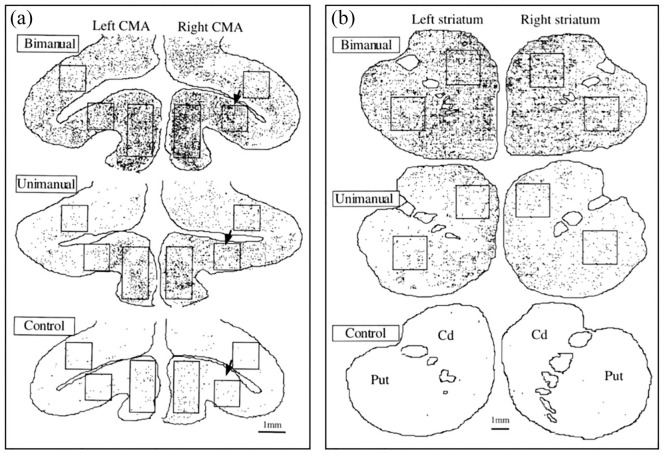
Reconstruction of individual frontal histological sections taken at the level of the cingulate motor cortex (CMA, a) and striatum (b), showing the location and density of Fos positive neurons (black dots). Mk-1 (bimanual task) sections are on top of each panel, whereas Mk-2 (unimanual task) and Mk-3 (no task) are in the middle and in the bottom, respectively. Squares and rectangles represent specific zones in which quantification was conducted on consecutive sections along the whole rostro-caudal axis in order to produce the quantitative data shown in Figure 3. Cd = caudate nucleus; Put = putamen. Note the strongly different FLI density across monkeys, with a substantial decrease of FLI from Mk-1 (top) to Mk-2 (middle) and from Mk-2 (middle) to Mk-3 (bottom). The arrows in panel A point to the 3 zones illustrated in the form of photomicrographs in Figure 1 (a-c).

**Figure 3.**
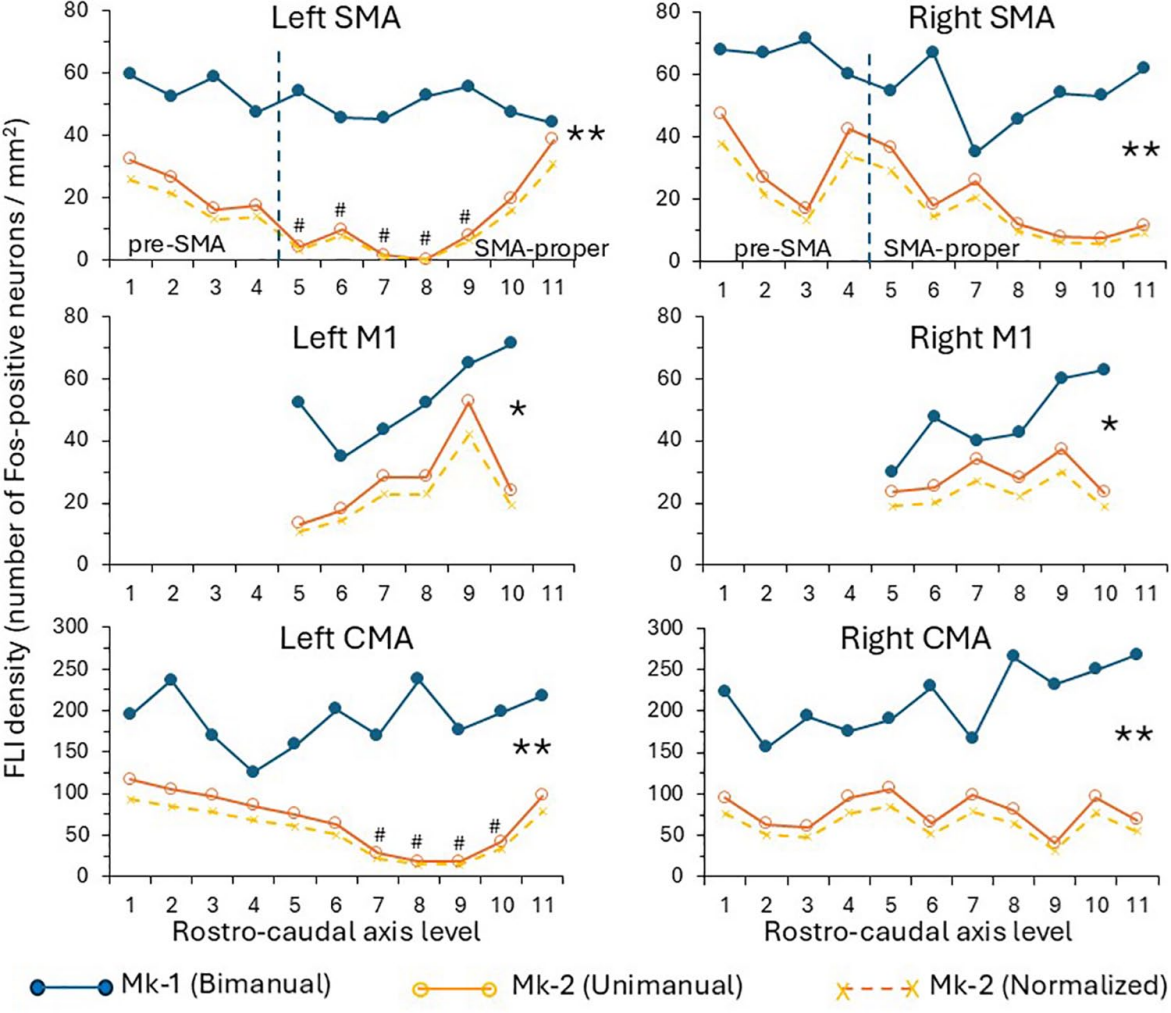
Quantitative data in the supplementary motor area (SMA), primary motor cortex (M1) and cingulate motor area (CMA), on the left hemisphere (left column) and on the right hemisphere (right column). The 2 solid lines curves represent the raw Fos data. The curves in the plot gives the density of FLI (ordinate) as a function of the rostro-caudal axis position of the corresponding individual sections (abscissa), going from rostral (left) to caudal (right). In M1 (hand area), the distance between 2 sections subjected to quantification was 0.7 mm, whereas it was 1.75 mm in SMA and CMA. In each plot, the top curve (close symbols in blue) is for Mk-1 (bimanual task) whereas the bottom curve (open symbols in brown) is for Mk-2 (unimanual task). The # symbols point to zones of the left SMA and CMA in Mk-2 in which a neuroanatomical tracer (BDA) was injected, partly and possibly obscuring the FLI. The vertical dashed lines in the top two plots represent the approximate limit between pre-SMA and SMA-proper, as defined in Liu et al.^89^ The stars on the right of each plot indicates the significance level of the differences between the 2 curves (Mk-1 vs Mk-2): * is for *p* < .05 and ** is for *p* < .01. The third curve in the bottom (brown crosses with dashed lines) represents the normalized data for Mk-2, after normalization using the LGN as a reference for comparison between Mk-1 and Mk-2 (see methods and results).

Under deep anesthesia, the monkeys were transcardially perfused with 0.9% saline solution (200 ml–300 ml) followed by 3 liters of fixative (4% phosphate-buffered paraformaldehyde), the brain was dissected, post-fixed for 3-5 days (in a mixture of fixative and 30% sucrose solution in phosphate buffer) and processed histologically, as previously reported.^18^ One of the five series of frontal histological sections (50 µm thick) was reacted with the Fos antibody (Figure 1a-c), from which the Fos-like positive neurons were plotted using a computer-assisted light microscope system^79^ (see Figures 1e and 2). The data files, including also the contours of brain structures, were imported into the NIH Image analysis software (1.58) allowing to establish the density of FLI (number of Fos positive neurons/mm^2^) in different brain regions (Figures 2 and 3). As the section sample areas to scan in M1, SMA, CMA and striatum were spatially restricted (see squares and rectangles in Figure 2), it was possible to visualize and chart every individual Fos positive neuron. As a consequence, it was thus feasible to use a quantification method based on “exhaustive plotting” instead of stereology, as also used in our laboratory for BDA anterograde tracing data for instance.^80-82^ Statistical comparisons either intra-animal between the two hemispheres or for each investigated brain area between the 2 experimental animals (Mk-1 vs Mk-2) were conducted based on the paired non-parametric Wilcoxon test, using an open-access software on-line (www.socscistatistics.com).

In addition to the statistical comparison between Mk-1 and Mk-2 applied to the raw data (calculated Fos density), it should be considered that the baseline Fos density (unrelated to the motor task) may be different between Mk-1 and Mk-2. For this reason, the Fos density was determined in non-motor areas (lateral geniculate nucleus and medial geniculate nucleus) in order to compare the baseline non-motor Fos density between Mk-1 and Mk-2. Then, the Fos density data were normalized based on that comparison, for comparison with the raw data (see results).

## Results

The FLI related to bimanual coordination was reported preliminarily and only qualitatively for M1 and SMA in a previous report,^18^ in the minimal form of an illustration of two individual histological sections taken at a single rostro-caudal level of M1 and SMA. The present study extends these early and preliminary data quantitatively (entire rostro-caudal extent) for M1 and SMA, as well as to additional brain areas (Figure 2), such as CMA and basal ganglia (striatum). Quantitative assessment of FLI density was conducted in central subregions of M1 and SMA along their rostro-caudal axis,^18^ (see Kermadi et al.^18^, Figure 13) more specifically in the rostral bank of the central sulcus in M1 corresponding to the so-called “new M1”^83^ and in the vertical portion of the frontal gyrus in SMA. In CMA, quantification was conducted in 3 representative subregions (Figure 2): the dorsal bank of the cingulate sulcus, the ventral bank of the cingulate sulcus and the vertical zone of the cingulate gyrus above the corpus callosum. In both Cd and Put, a central zone was delineated to conduct the quantification (Figure 2). Overall, in the 5 brain areas quantified, the restricted zones for quantification (rectangles as illustrated in Figure 2) were delineated so that they were reproducibly present and stable all along the considered rostro-caudal axis.

As shown in Figure 1a-c, corresponding to a representative sample, FLI was denser in a selected region of the ventral CMA in Mk-1 (bimanual performance; Figure 1a) than in Mk-2 (unimanual performance; Figure 1b), itself exhibiting a denser FLI than in a control monkey (Mk-3; no motor performance; Figure 1c). The increased FLI observed in Mk-1 (bimanual task performance) and in Mk-2 (unimanual task performance before euthanasia), as compared to the control monkey Mk-3 (no task), supports the concept that repetitive and intense voluntary motor activity triggered the immediate early gene c-fos in brain motor regions, leading to the production of the Fos protein detected immuno-histologically (Figure 1a-c; Figure 2a and b). The dramatic contrast of FLI density between the control monkey (MK-3) on one hand and the 2 experimental monkeys (Mk-1 and Mk.2) on the other hand is quantitatively shown in Table 1, in which ratios of FLI density were calculated. Across the 5 regions of interest, the ratios of FLI densities in Mk-2 exceeding the corresponding ones in the control monkey ranged from 6 to 113 times. For Mk-1, the corresponding range in comparison to the control monkey was 12 to 396 times.

**Table 1.** Ratio of Fos labelling density in the Unimanual (middle line) and Bimanual (bottom line) monkeys, with respect to the Fos labeling density in the control monkey (Mk-3). For instance, Fos labelling in M1 was 38 times denser in the Uni Mk-2 than in the control Mk-3. Similarly, it was 62 times denser in M1 in the BIM Mk-1 than in the control Mk-3.

Monkey ID	M1	SMA	CMA	Put	Cd
Control Mk-3	1	1	1	1	1
UNI Mk-2	38	7.5	6	113	70
BIM Mk-1	62	29.1	12.4	396	269

**Figure 4.**
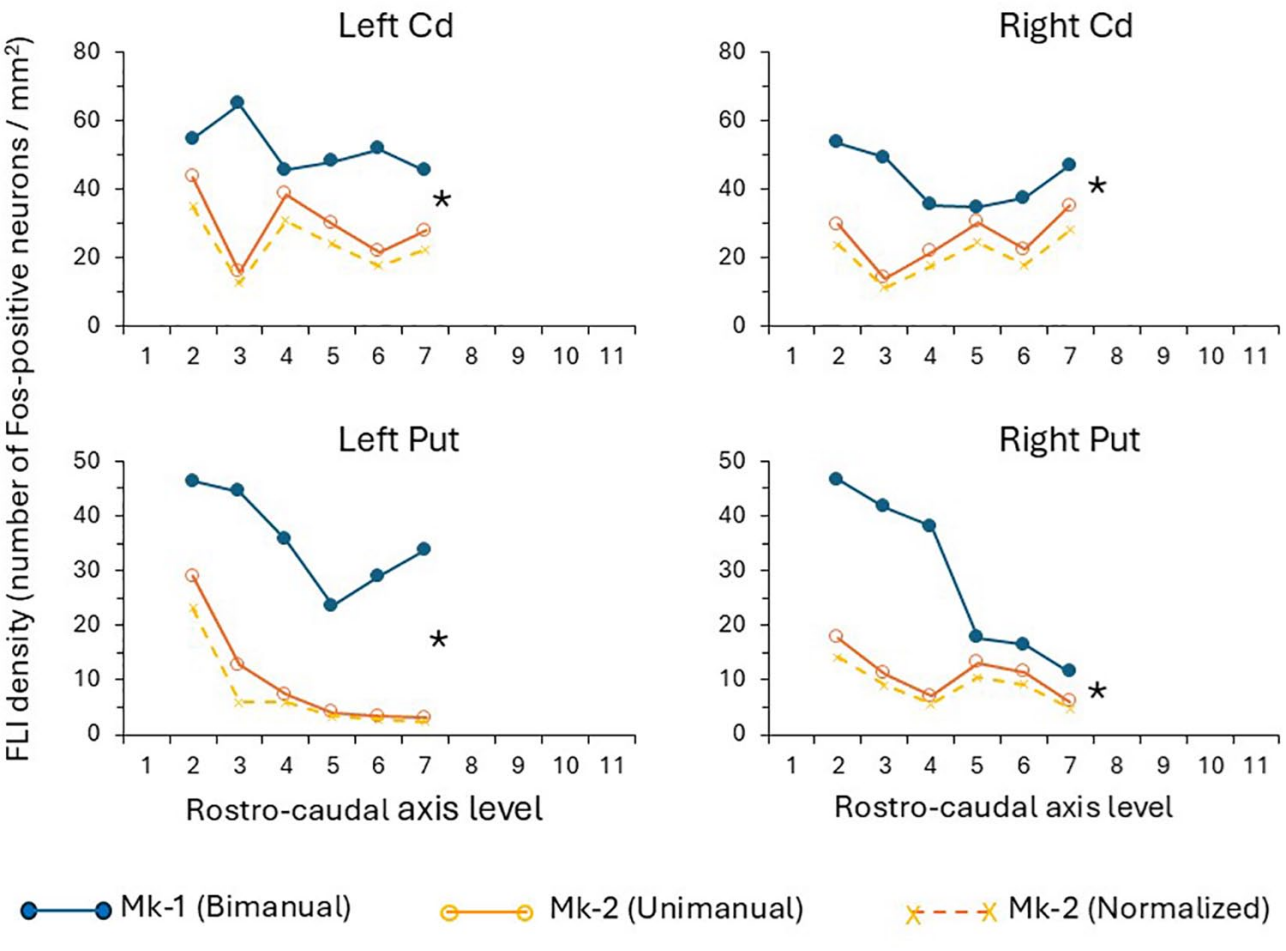
Quantitative data in the caudate nucleus (Cd) and putamen (Put), on the left hemisphere (left column) and on the right hemisphere (right column). The 2 solid lines curves represent the raw Fos data. The curves in the plot gives the density of FLI (ordinate) as a function of the rostro-caudal axis position of the corresponding individual sections (abscissa), going from rostral (left) to caudal (right). The distance between 2 sections subjected to quantification was 1.75 mm in Cd and Put. In each plot, the top curve (close symbols in blue) is for Mk-1 (bimanual task) whereas the bottom curve (open symbols in brown) is for Mk-2 (unimanual task). The stars on the right of each plot indicates the significance level of the differences between the 2 curves (Mk-1 vs Mk-2): * is for *p* < .05. The third curve in the bottom (brown crosses with dashed lines) represents the normalized data for Mk-2, after normalization using the LGN as a reference for comparison between Mk-1 and Mk-2 (see methods and results).

As illustrated qualitatively for CMA and the striatum (Figure 2a and b), the denser FLI observed in Mk-1 as compared to Mk-2 suggests that more medium-size and small neurons were active in CMA, caudate nucleus (Cd) and putamen (Put) when the monkey performed a bimanual task (Mk-1) as compared to a unimanual task (Mk-2). To support this conclusion, in the same two monkeys, FLI was assessed in non-motor brain regions, such as the lateral (LGN) and medial (MGN) geniculate nuclei of the thalamus (Figure 1e). In contrast to M1, SMA, CMA, Cd and Put, FLI was comparable between Mk-1 and Mk-2 in LGN and MGN (Figure 1e), consistent with the conclusion that the FLI difference between Mk-1 and Mk-2 is most probably motor task related. Further support to this conclusion was provided by the observation that FLI in another non-motor brain region, namely the mamillary nuclei, was similar in Mk-1 and Mk-2 (not shown), which was similar also to FLI in the control monkey (Mk-3; not shown). FLI density quantification conducted in LGN and MGN for Mk-1 and Mk-2 showed that it was similar in the MGN (209 and 210 FLI positive neurons/mm^2^ in Mk-1 and Mk-2, respectively). In LGN, the FLI density was 51 and 63 FLI positive neurons/mm^2^ in Mk-1 and Mk-2, respectively. These FLI values in LGN were used to normalize the FLI data in the 5 brain regions of interest, for further comparison between Mk-1 and Mk-2 (see below, as well as Figures 3 and 4).

The FLI in Mk-1 and Mk-2 was quantified in selected regions of M1, SMA, CMA, Cd and Put, for inter-animal comparison, as well as for interhemispheric comparison, at consecutive sections all along the rostro-caudal axis of these motor regions. These quantitative FLI data are shown in Figures 3 and 4, separately for the left and right hemispheres, in Mk-1 (bimanual task) and in Mk-2 (unimanual task). Within each animal (Monkeys 1-2), and for each region investigated (M1, SMA, CMA, Cd, and Put), the FLI densities at the same consecutive rostro-caudal levels did not show any statistical difference between the left and right hemispheres (paired non-parametric comparison with the Wilcoxon-test: *p* > .05). The interhemispheric symmetrical FLI distribution and density appear clearly in Figures 2–4.

In all motor areas investigated (SMA, M1, CMA, Cd, Put), FLI was systematically higher at all rostro-caudal coordinates in Mk-1 (bimanual task; upper curves with filled blue symbols in Figures 3 and 4) than at the same coordinate in Mk-2 (unimanual task; lower curves with open brown symbols in Figures 3 and 4). In all 5 investigated brain areas, these Fos density differences between Mk-1 and Mk-2 were statistically significant, based on the paired non-parametric Wilcoxon test (* for *p* < .05 and ** for *p* < .01 in Figures 3 and 4). The difference between Mk-1 and Mk-2 was most prominent in SMA, CMA and Put, somewhat less in M1 and Cd, although also systematic in the latter two regions (Figures 3 and 4). Interestingly, the quantitative data appear comparable on both hemispheres in the five motor areas investigated, an observation expected for the bimanual task (Mk-1), but not for the unimanual task (Mk-2). Possible interpretations of an interhemispheric symmetry of FLI distribution also for the unimanual task (Mk-2) are provided below in the discussion section. Considering the absolute FLI density numbers, it appears that CMA exhibits a clearly higher FLI density than the other motor areas (M1, SMA, Cd, Put), irrespectively of the task (bimanual or unimanual). In 4 of the 5 motor areas investigated (Figures 3 and 4), there was no strong FLI change along the rostro-caudal axis (SMA, M1, CMA, Cd); in contrast, FLI was clearly higher rostrally than caudally in Put (Figure 4). Although SMA was demonstrated to be composed of two functionally and connectionally distinct rostral and caudal parts,^84-91^ the clear FLI difference between Mk-1 and Mk-2 was present in both its rostral part (pre-SMA) and in its caudal part (SMA-proper; Figure 3). As argued below in the discussion section, the systematic FLI difference related to the motor paradigm (bimanual versus unimanual) is believed to reflect, at least in a large part, an excess of neural activity required to coordinate both forelimbs in time and space when performing the bimanual reach and grasp prehension task.

In order to take into account a possible inter-individual difference between Mk-1 and Mk-2 with respect to a baseline non-motor FLI density, a normalization of the data was conducted, using the LGN as a non-motor area of reference. As mentioned above, FLI in LGN was about 20% higher in Mk-2 than in Mk-1 (63 vs 51, respectively). As a consequence, to take into account such an inter-individual difference, the FLI densities in Mk-2 were normalized by multiplying them by the ratio 51/63, yielding the brown dashed curves in Figures 3 and 4 (x symbols). The impact of such normalization is to increase the differences between Mk-1 and Mk-2, observed on the raw data. Note that a normalization based on the MGN would have no impact, as FLI was similar in the MGN for Mk-1 and Mk-2 (see above).

## Discussion

Very few studies used Fos to map neuronal activity in non-human primates, either in response to external stimuli,^92,93,94^ brain electric stimulation^68,95^ or in relation to a specific behavior,^92,96,97,98^ those conducted in macaques are indicated above with an asterisk. The present study represents most likely a unique Fos mapping study conducted in (two) macaque monkeys in relation to a controlled motor task requiring coordination of the two hands, associated with electrophysiological single neuron recordings derived in the awake state in the same animals and for the same motor performance.

In the unimanual left hand only task (Mk-2), all manipulations of the drawer (reaching, knob grasping, drawer pulling, pellet grasping and transport back to the mouth) were executed exclusively with the left hand, while the right hand remained still on the starting pad. The drawer was mechanically kept open by the set-up, so that the pellet could be retrieved with the same left hand. However, maintaining the right hand still on the starting pad involves some motor control via activation of several axial, proximal and distal muscles, while other muscles need to be inhibited, both on the right body side. In the bimanual task, while the right hand was used to grasp the pellet, the left hand was solicited in order to keep the drawer open. Comparing the two tasks, the resting activity of the right hand on the starting pad in the unimanual left hand version is likely to be roughly comparable to the activity of the left hand also in a resting state while keeping the drawer open in the bimanual version. The pellet grasping phase with the left hand in the unimanual sequence is similar to that executed with the right hand in the bimanual sequence. In sum, the overall motor activity of the two individual arms in the two tasks are fairly comparable, except that the bimanual task involves an additional coordination between the two arms/hands in space and in time, in particular the precise programming of what the left hand is doing while the right hand is performing a totally different movement sequence (including the prevention of performing mirror movements). As a consequence of a fairly comparable motor activity generated by each individual hand before euthanasia in Mk-1 and Mk-2, it is plausible that the stronger FLI observed in M1, SMA, CMA, Put and Cd for Mk-1 as compared to Mk-2 (Figures 2, 3 and 4) may reflect the excess of brain processing needed to plan and control a bimanual coordinated task, as compared to a unimanual comparable task. The increased FLI related to bimanual control is thus widespread cortically (M1, SMA, CMA) and subcortically (striatum), in line with previous electrophysiological investigations in the same 2 monkeys which reported the presence of “bimanual” neurons in M1, SMA, CMA, PMd, AIP and the basal ganglia.^18,19,33^ However, electrophysiology (single neuron recording) or FLI reflect the activity of most likely distinct neuronal populations, large versus small/medium size cells, respectively. Based on different bimanual paradigms, other electrophysiological or imaging studies reached a similar conclusion supporting the notion of a widely spread neural control of interlimb coordination, both in non-human primates and humans.^15,16,21-30,32,34-42,45,47-52,99^

An unexpected finding in the present study was the absence of a hemispheric lateralization of FLI density in the unimanual task (Mk-2; Figures 2, 3 and 4). Electrophysiological data revealed a strong predominance of neurons mostly active when the monkey performs unilateral movements with the contralateral arm as compared to the ipsilateral hand, in most “motor” cortical areas.^15,18,19,21,36,47,50^ Why then such lateralization did not appear in the FLI of Mk-2 while performing the task unimanually (Figures 2-4)? In electrophysiological recordings, bias towards large neurons makes that layer V corticospinal neurons are likely to contribute to a substantial extent to the recorded neuronal population; as they project predominantly to the contralateral spinal cord, ^4,100-103^ then a lateralization towards the contralateral forelimb is commonly observed. In contrast, the small and medium size neurons in motor cortical areas, which are revealed by the Fos protein and do not project at long distance, are most probably less lateralized functionally. Furthermore, the lack of interhemispheric FLI asymmetry in Mk-2 (unimanual task) may also be due to small, uncontrolled movement of the right arm during the task, to the brain activity needed to keep the right arm motionless on the resting pad, as well as the fact that proximal and axial muscles of the arm, also involved in the present task, are also controlled ipsilaterally, for instance via uncrossed corticospinal projections. ^4,104-106^ Finally, in between the trials (while eating the reward), Mk-2 (unimanual task) was free to make movements with the right forelimb. Nevertheless, the absence of lateralization of FLI density in Mk-2 remains a bit unexpected, raising questions about the sensitivity of the c-fos method, its restrictive selectivity for a few sub-populations of cells, not or only partially representative of the overall neural activity. Furthermore, representing a further limitation, this result may be linked to the current behavioral paradigm (reach and grasp drawer task), asking for comparisons with future c-fos studies based on different bimanual versus unimanual motor paradigms.

The notion that SMA is “the brain center” specifically controlling bimanual coordination (referring here to its historical context several decades ago) is challenged by the present FLI data and earlier electrophysiological data,^18,19,33^ rather supporting the concept of a widely distributed network including at least M1, PM, SMA, CMA, AIP and the striatum. Extensive projection systems interconnect those structures homolaterally,^86,107-121^ as well as between the left and right hemispheres. ^79,89,106,107,122-126^

As far as SMA is concerned, a particular role seems to rather be a specific responsibility in the initiation of voluntary movements, especially when self-paced.^17,127^ Indeed, besides a deficit in task initiation, a reversible unilateral inactivation of SMA did not impair the execution of the bimanual reach and grasp drawer task per se, that is, the reaching, pulling, grasping and withdrawing movement phases, while a unilateral reversible inactivation of M1 strongly impaired the contralateral arm for reaching and grasping.^17,128^,^129^

An obvious limitation of the present study, inherent to nonhuman primate invasive investigations, is the small number of individuals involved (*n* = 3), with only two monkeys performing the behavioral tasks (1 control monkey in addition). However, it is common to restrict to 2 monkeys studies combining complex behavioral training with single unit recordings in the awake state. In the present case, the two monkeys were the same from which single unit recordings were derived from M1, SMA, CMA, and AIP^18,19^ and FLI assessed in the same cortical areas, plus the striatum, for the same bimanual versus unimanual tasks. Single unit recordings conducted in monkeys performing a sophisticated motor task were often limited to a single or very few brain areas investigated whereas the present FLI study allows a comprehensive functional survey of the whole brain in an individual monkey. This is also the case of metabolic functional survey with 2DG (2-deoxyglucose) for instance,^130-132^ although FLI has the advantage to provide cellular resolution over 2DG (but FLI is biased to small/medium size neurons). Nevertheless, recent electrophysiological advances using chronic multiple electrode arrays (e.g. 32-128 micro-electrodes Utah arrays) now offer the possibility to conduct in parallel in the same animal electrophysiological investigations in several cortical areas.^133,134^ However, it remains that some cortical areas (e.g. CMA, parts of the SMA, the “new M1 area” in the rostral bank of the central sulcus, or subcortical regions such as basal ganglia or thalamus) are not easy if not impossible at present to access with multiple micro-electrode arrays, which are more suitable to be placed on cortical areas located at the brain surface. Functional mapping techniques like Fos thus remain pertinent approaches to investigate brain regions difficult to access with chronic multi- micro-electrode probes. A further limitation in the interpretation of the present Fos data is that the two experimental monkeys (Mk-1 and Mk-2) were subjected to an overall long training period and electrophysiological investigation period (more than 2 years), which may have induced long-term plasticity effects, possibly influencing the FLI pattern. However, the training and recording periods were comparable for the 2 monkeys and conducted in parallel over the same time course. In the context of animal protection, and reduction of the number of monkeys involved, it would seem inadequate to train monkeys to a sophisticated behavioral task, followed by a single final behavioral session with euthanasia in order to assess FLI only, without combination with preceding electrophysiology for instance.

Two brain regions exhibited a particularly strong and/or a well spatially segregated zone of FLI, CMA and the rostral part of Put, respectively (Figures 2, 3 and 4). Interestingly, these 2 areas (CMA and rostral Put) are strongly associated via a preferential corticostriate projection of CMA onto the rostral Put.^116^ Furthermore, in Put FLI was most dense in its ventral zone (Figure 2), which corresponds somatotopically to a finger region (proximal part located more dorsal),^117^ in line with a bimanual task strongly involving distal forelimb muscles. The strong FLI in the striatum observed in MK-1 in relation to bimanual motor act (Figures 2 and 4) is consistent with bimanual coordination deficits reported for Parkinson’s disease patients.^135-138^

C-fos immunocytochemistry does not label the entire neuronal cell body, but only the nucleus of the Fos positive cells. As a consequence, it was not possible to identify individually and directly the cellular type of each Fos positive neurons, as well as to quantify FLI across neuron size, representing an obvious limitation in the interpretation of the current data. The bias of FLI towards “small and medium” size neurons has been repeatedly reported in the auditory system.^60-64,72-76^ For instance, in both the ventral cochlear nucleus and the ventral division of the MGN, where large size principal neurons are dominant, FLI was sparse, on the contrary to dense FLI observed in small size neurons’ regions, like the granule cell domain in the cochlear nucleus. In the present Fos study focused on motor cortical areas (M1, SMA, CMA), if the majority of large pyramidal neurons would be Fos positive, then an increased FLI density would be present at the corresponding position in the cortical layers III and V. This is not what was observed, as the Fos labeling was rather homogeneous across cortical layers (see Figure 2 for CMA and Figure 13 in our previous report^18^). This observation thus suggests that the largest cortical neurons (pyramidal cells) were not detected for the most part with the current Fos immunoreactivity functional mapping technique, in contrast to smaller size neurons (referred to as “small and medium size neurons” above). The issue of Fos labelling with respect to neuronal size may also depend on the CNS location and/or species: in the spinal cord of the rat, following a locomotion task, about 20% of motoneurons (large neurons) in lamina IX were Fos positive.^71^

## Conclusion and future directions

Using the FLI functional mapping method, complementary to single neuron electrophysiology, both providing cellular resolution but addressing largely different subpopulations of neurons in terms of size, the present study confirms the notion of a widely distributed network of brain regions controlling interlimb coordination. FLI allowed here to test subpopulations of small and medium size neurons, which tend to be less often sampled by electrophysiological techniques. FLI also focuses more on local neurons than on long distance projecting neurons (cortical pyramidal cells of layers III and V), again privileged with single unit recording techniques. The increased FLI observed in M1, SMA, CMA and striatum in relation to bimanual motor act suggests that a complex neural processing underlying interlimb coordination is not only reflected in the activity of large size, long distance projecting neurons (sampled by electrophysiology), but also in the activity of small and medium size neurons involved in more local information processing. In the future, the present study may encourage researchers who have completed electrophysiological investigations in behaving monkeys to terminate their study with an acute terminal FLI investigation before euthanasia.

## Significance Statement

- Monkeys performed either a unimanual or a bimanual reach and grasp drawer task, which triggered motor related activation of the immediate early gene c-fos.
- The activation of c-fos was detected immunocytochemically in brain motor areas on histological sections using an antibody against the protein Fos.
- Fos-like immunoreactivity (FLI) was significantly stronger when the monkey performed the task bimanually as compared to unimanually, in M1, SMA, CMA, and striatum.
- The bimanually related increase of FLI may possibly reflect an excess of neural activity underlying interlimb coordination in small and medium size neurons.

## Notes

i. All experiments in macaque monkeys were covered by Swiss official veterinary authorizations (see for detail the related publications from our laboratory listed in the introduction section: Background and Aim). The author has carefully read and adhered to the ARRIVE guidelines. Monkeys had free access to water at all times in their common housing facility (at that time under an ancient regulation, 2-5 monkeys in a 15 m^3^ detention room) and they were not food restricted either. The rewards received during the behavioral session in the morning were their first access to food daily, followed by additional food provided later during the day (cereals, fruits).
